# Supplementary material for: A primary school-based dengue solution model for post-COVID-19 in southern Thailand: Students understanding of the dengue solution and larval indices surveillance system
Source: PLoS One. 2024 Dec 31;19(12):e0313171. doi: 10.1371/journal.pone.0313171 (PMC11687803; doi:10.1371/journal.pone.0313171)
Supplement: S1 File — (DOCX) [file pone.0313171.s002.docx]

**Supplement 2: Leader student to understanding dengue solution (UDS) and understanding larval indices surveillance system (ULISS)**

**1.2 Understanding dengue solution (UDS) of Leader student among four primary schools**

The correct UDS items were increasing in post-test, non-item in school A, and B, eight items in school C, and an item in school D. The comparison total UDS of leader student, there were six items in post-test were increasing different significant from pre-test of total leader students (P<0.05) such as 1, 2, 3, 4, 6, 7, 8, and 10 showed Odd ratio (OR) 4.67, 2.62, 4.87, 2.56, 8.47, 3.29, 2.16, and 3.67 respectively. Two items number 5, and 9 were no increasing significantly statistic (P> 0.05)**.**

**Table 3** Comparison correct answer of understanding dengue solution of leader student among four primary schools pre and post intervention

| **Leader student’s UDS** | **Time** | **Correct answer**  ***n(%)*** | | | | **Total of school** | **OR**  **of total** | **95%CI** | |
| --- | --- | --- | --- | --- | --- | --- | --- | --- | --- |
|  |  | **A** | **B** | **C** | **D** |  |  | **Lower** | **Upper** |
| 1. If a patient has a high fever for 2 to 7 days, petechiae, and a painful enlargement of the liver, the patient is showing signs of dengue infection. | Pre^ref^ | 12  (70.58) | 7  (87.50) | 13  (54.17) | 7  (63.63) | 39  (65.00) | 4.67*** | 1.94 | 12.29 |
|  | Post | 20  (83.33) | 16  (88.89) | 22  (100.00) | 13  (86.67) | 71  (89.87) |  |  |  |
|  | $p$ | 0.450^ns(a)^ | 1.000^ns(a)^ | 0.001^***^ | 0.348^ns(a)^ |  |  |  |  |
| 2. If the patient presents signs and symptoms of dengue, then the patient is showing signs of a dengue viral infection. | Pre^ref^ | 6  (35.29) | 3  (21.4) | 6  (25.0) | 5  (45.45) | 20  (33.33) | 2.62** | 1.31 | 5.36 |
|  | Post | 7  (29.17) | 11  (78.6) | 18  (81.82) | 9  (60.00) | 45  (56.96) |  |  |  |
|  | $p$ | 0.940^ns^ | 0.401^ns(a)^ | 0.000*** | 0.736 |  |  |  |  |
| 3. If a person living in a high dengue risk area is infected with one dengue serotype, they may have lifelong immunity to that strain. However, they would still be vulnerable to other serotypes and could, thus, be infected with the other dengue serotypes later in life. | Pre^ref^ | 8  (47.06) | 6  (27.3) | 9  (37.50) | 6  (54.55) | 29  (48.33) | 4.87*** | 2.29 | 10.83 |
|  | Post | 17  (70.83) | 16  (72.7) | 19  (86.36) | 13  (86.67) | 65  (82.28) |  |  |  |
|  | $p$ | 0.225 ^ns^ | 0.563 ^ns(a)^ | 0.002** | 0.095^ns(a)^ |  |  |  |  |
| 4. If a patient is protected from female *Aedes aegypti* bites, they will be safe from dengue. | Pre^ref^ | 14  (82.35) | 8  (100.00) | 14  (58.33) | 9  (81.82) | 45  (75.00) | 2.56* | 1.04 | 6.64 |
|  | Post | 21  (87.50) | 15  (83.33) | 21  (95.45) | 13  (86.67) | 70  (88.61) |  |  |  |
|  | $p$ | 0.679 ^ns(a)^ | 0.529 ^ns(a)^ | 0.009** | 1.000 ^ns(a)^ |  |  |  |  |
| 5. If a patient has a high fever for 2–7 days, nausea, vomiting, and possible abdominal pain, the patient is in the fever stage. | Pre^ref^ | 5  (29.41) | 2  (25.00) | 13  (51.17) | 4  (36.63) | 24  (40.00) | 1.39 ^ns^ | 0.70 | 2.76 |
|  | Post | 2  (8.33) | 8  (44.44) | 16  (72.72) | 12  (80.00) | 38  (48.10) |  |  |  |
|  | $p$ | 0.105 ^ns(a)^ | 0.420^ns(a)^ | 0.319 ^ns^ | 0.043*^(a)^ |  |  |  |  |
| 6. If a patient with dengue hemorrhagic fever presents pain at the right lower costal margin, they are showing signs of hepatomegaly. | Pre^ref^ | 2  (11.76) | 4  (50.00) | 5  (20.83) | 5  (45.45) | 16  (26.67) | 8.47*** | 3.99 | 18.89 |
|  | Post | 17  (70.83) | 14  (77.78) | 18  (81.82) | 11  (73.33) | 60  (75.95) |  |  |  |
|  | $p$ | 0.001*** | 0.197^ns(a)^ | 0.000*** | 0.115 ^ns(a)^ |  |  |  |  |
| 7. If a patient with dengue hemorrhagic fever presents signs of shock from leakage of plasma, they will have poor tissue perfusion, weak pulse, and narrowed pulse pressure. | Pre^ref^ | 3  (21.43) | 4  (50.00) | 8  (33.33) | 7  (63.64) | 22  (36.67) | 3.29*** | 1.64 | 6.75 |
|  | Post | 10  (41.67) | 14  (77.78) | 19  (86.36) | 9  (60.00) | 52  (65.82) |  |  |  |
|  | $p$ | 0.198^ns^ | 0.197^ns(a)^ | 0.001*** | 1.000 ^ns(a)^ |  |  |  |  |
| 8. If your neighbor presents signs of poor tissue perfusion, weak pulse, and clammy skin, you need to send them to hospital. | Pre^ref^ | 6  (35.29) | 4  (50.00) | 12  (50.00) | 7  (63.64) | 29  (48.33) | 2.16* | 1.09 | 4.37 |
|  | Post | 10  (41.67) | 14  (77.78) | 19  (86.36) | 10  (66.67) | 53  (67.09) |  |  |  |
|  | $p$ | 0.931^ns^ | 0.197^ns(a)^ | 0.021* | 1.000^ns(a)^ |  |  |  |  |
| 9. Dengue patients should avoid consuming aspirin or non-steroidal anti-inflammatory drugs because they may cause gastritis and subsequent massive gastrointestinal or hepatic injury. | Pre^ref^ | 4  (36.4) | 0  (0.00) | 3  (12.50) | 5  (45.45) | 12  (20.00) | 1.53 ^ns^ | 0.69 | 3.52 |
|  | Post | 7  (63.6) | 4  (22.22) | 3  (13.64) | 8  (53.33) | 22  (27.85) |  |  |  |
|  | $p$ | 0.736 ^ns(a)^ | 0.277 ^ns(a)^ | 1.000 ^ns(a)^ | 1.000 ^ns^ |  |  |  |  |
| 10. If your neighbor presents high fever on day 1, you give one paracetamol every 6 hr and a tepid sponge bath. | Pre^ref^ | 2  (23.53) | 1  (12.50) | 8  (33.33) | 8  (72.73) | 19  (31.67) | 3.67*** | 1.82 | 7.63 |
|  | Post | 9  (37.50) | 10  (55.56) | 20  (83.33) | 11  (73.33) | 50  (63.29) |  |  |  |
|  | $p$ | 0.085 ^ns(a)^ | 0.084 ^ns(a)^ | 0.000*** | 1.000 ^ns(a)^ |  |  |  |  |

*Total student of level 4, 5, and 6 such as A: 94 students; B: 43 tudents; C: 42 students; D: 41 students*

*p* *: p-value of Chi-square test used unless otherwise noted.* ^(a)^*Fisher’s exact test used. ^***^p<0.001 ^**^p<0.01 ^*^p<0.05 ^ns^ Non significant*

*A, B, C and D: primary school; OR of total: Odd ratio; 95% CI: 95% Confidence interval*

*n of A=*

**1.2 Understanding larval indices surveillance system of leader students among four primary schools**

The correct UDS items were increasing in post-test, non-item in school A, and B, eight items in school C, and an item in school D. The comparison total UDS of leader student, there were six items in post-test were increasing different significant from pre-test of total non-leader students (P<0.05) such as 1, 2, 3, 4, 6, 7, 8, and 10 showed 4.67, 2.62, 4.87, 2.56, 8.47, 3.29, 2.16, and 3.67 respectively. Two items number 5, and 9 were no increasing significantly statistic (P> 0.05).

**Table 4** Comparison correct answer of understanding larval indices surveillance system among four primary schools pre and post intervention

| **Leader student’s ULISS** | **Time** | **Comparison correct *answer***  ***n(%)*** | | | | **Total of school** | **OR**  **of total** | **95%CI OR**  **of total** | |
| --- | --- | --- | --- | --- | --- | --- | --- | --- | --- |
|  |  | **A** | **B** | **C** | **D** |  |  | Lower | Upper |
| 1. The number of larvae of female *Aedes aegypti* in the areas are indicated by the larval indices. | Pre^ref^ | 0  (0.00) | 1  (12.50) | 5  (20.83) | 1  (9.09) | 7  (11.67) | 2.20^ns^ | 0.87 | 6.11 |
|  | Post | 3  (12.50) | 4  (22.22) | 5  (22.73) | 6  (40.00) | 18  (22.78) |  |  |  |
|  | $p$ | 0.254^ns(a)^ | 1.000^ns(a)^ | 1.000^ns(a)^ | 0.178^ns(a)^ |  |  |  |  |
| 2. Through the container index (CI), the value for identifying a dengue outbreak is calculated. Then, the number of water containers and the number of water containers infested with larvae are surveyed. | Pre^ref^ | 6  (35.29) | 3  (37.50) | 6  (25.00) | 6  (54.55) | 21  (35.00) | 1.63^ns^ | 0.82 | 3.29 |
|  | Post | 10  (41.67) | 5  (27.78) | 14  (63.64) | 8  (53.33) | 37  (46.84) |  |  |  |
|  | $p$ | 0.931^ns^ | 0.667^ns(a)^ | 0.019* | 1.000^ns^ |  |  |  |  |
| 3. The participation of family leaders in the survey for larval indices in and out of the household every 7 days is key. | Pre^ref^ | 4  (23.52) | 4  (50.00) | 8  (33.33) | 6  (54.55) | 22  (36.67) | 5.01*** | 2.44 | 10.63 |
|  | Post | 13  (54.17) | 13  (72.22) | 20  (90.91) | 13  (86.67) | 59  (74.68) |  |  |  |
|  | $p$ | 0.101^ns^ | 0.382^ns(a)^ | 0.000*** | 0.095^ns(a)^ |  |  |  |  |
| 4. Insecticide *Temephos* sand granulates may be used to eliminate larvae, but not to eliminate mosquito eggs. | Pre^ref^ | 6  (35.29) | 2  (25.00) | 2  (8.33) | 0  (0.00) | 10  (16.67) | 2.42* | 1.08 | 5.79 |
|  | Post | 9  (37.50) | 2  (11.11) | 10  (45.45) | 5  (33.33) | 26  (32.91) |  |  |  |
|  | $p$ | 1.000^ns^ | 0.563^ns(a)^ | 0.011* | 0.053^ns(a)^ |  |  |  |  |
| 5. You suggest the use of lotion in the neighborhood as a prevention of mosquito bites. | Pre^ref^ | 4  (23.53) | 4  (50.00) | 5  (20.83) | 4  (36.36) | 17  (28.33) | 5.69*** | 2.75 | 12.22 |
|  | Post | 16  (66.67) | 14  (77.78) | 15  (68.18) | 10  (66.67) | 55  (69.62) |  |  |  |
|  | $p$ | 0.016* | 0.197^ns(a)^ | 0.003** | 0.257^ns^ |  |  |  |  |
| 6. If people in the village have dengue, you suggest for all to prevent dengue by eliminating mosquito breeding sites and endeavoring to diminish the scores for the larval indices around the house. | Pre^ref^ | 5  (29.41) | 3  (37.50) | 10  (47.67) | 2  (18.18) | 20  (33.33) | 1.36^ns^ | 0.67 | 2.77 |
|  | Post | 5  (20.83) | 5  (27.78) | 17  (77.27) | 5  (33.33) | 32  (40.51) |  |  |  |
|  | $p$ | 0.714^ns(a)^ | 0.667^ns(a)^ | 0.032* | 0.658^ns(a)^ |  |  |  |  |
| 7. You suggest eliminating mosquito larvae by cleansing and scrubbing the edge of the container over the area that used to have water. | Pre^ref^ | 2  (11.76) | 3  (37.50) | 4  (16.67) | 2  (18.18) | 11  (18.33) | 1.92^ns^ | 0.87 | 4.50 |
|  | Post | 3  (12.50) | 3  (16.67) | 10  (45.45) | 8  (53.33) | 24  (30.38) |  |  |  |
|  | $p$ | 1.000^ns(a)^ | 0.330^ns(a)^ | 0.072^ns^ | 0.109^ns(a)^ |  |  |  |  |
| 8. You suggest putting the water container upside down until the larvae can be seen. You also check the container again, considering that the lifespan of a mosquito is 1–5 years. | Pre^ref^ | 4  (23.53) | 2  (25.00) | 2  (8.33) | 2  (18.18) | 10  (16.67) | 8.86*** | 4.01 | 21.20 |
|  | Post | 12  (50.00) | 13  (72.22) | 16  (72.73) | 10  (66.67) | 51  (64.57) |  |  |  |
|  | $p$ | 0.165 ^ns^ | 0.038*^(a)^ | 0.000*** | 0.040* |  |  |  |  |
| 9. If the water container capacity is 100 L, we can use red lime in the container. | Pre^ref^ | 3  (17.65) | 2  (25.00) | 8  (33.33) | 1  (9.09) | 14  (23.33) | 10.82*** | 4.99 | 24.89 |
|  | Post | 17  (70.83) | 13  (72.22) | 21  (95.45) | 10  (66.67) | 61  (77.22) |  |  |  |
|  | $p$ | 0.002** | 0.038*^(a)^ | 0.000*** | 0.005**^(a)^ |  |  |  |  |
| 10. The larval indices surveillance system must be documented on the 25th of every month in the “Violet book”. | Pre^ref^ | 2  (11.76) | 0  (0.00) | 2  (8.30) | 1  (9.09) | 5  (8.33) | 5.52*** | 2.11 | 17.60 |
|  | Post | 9  (37.50) | 7  (38.39) | 10  (45.50) | 1  (6.67) | 27  (34.62) |  |  |  |
|  | $p$ | 0.085^ns(a)^ | 0.062^ns(a)^ | 0.011* | 1.000^ns(a)^ |  |  |  |  |

*Total student of level 4, 5, and 6 such as A: 94 students; B: 43 tudents; C: 42 students; D: 41 students*

*p* *: p-value of Chi-square test used unless otherwise noted.* ^(a)^*Fisher’s exact test used. ^***^p<0.001 ^**^p<0.01 ^*^p<0.05 ^ns^ Non significant****;*** *A, B, C and D: primary school; OR of total: Odd ratio; 95% CI: 95% Confidence interval*
