# Supplementary material for: A primary school-based dengue solution model for post-COVID-19 in southern Thailand: Students understanding of the dengue solution and larval indices surveillance system
Source: PLoS One. 2024 Dec 31;19(12):e0313171. doi: 10.1371/journal.pone.0313171 (PMC11687803; doi:10.1371/journal.pone.0313171)
Supplement: Attachment — Submitted filename: Response Letter.docx [file pone.0313171.s004.docx]

PLOS ONE Journal.

29th September, 2024.

Dear Prof. Rajib Chowdhury,

We would like to thank you and the respected reviewers for their thoughtful evaluation of our manuscript (ID Number, **PONE-D-24-29223**) entitled ***“******A primary school-based dengue solution model for post-COVID-19 in southern Thailand: Students understanding of the dengue solution and larval indices surveillance system”***. Please find our revised manuscript (with highlighted changes), which we believe is substantially strengthened now that we have incorporated reviewers’ recommendations.

**Journal Requirements**

We have thoroughly reviewed the journal requirements and made changes accordingly.

**Response to Reviewers’ comments**

**Response to Reviewer 1 comments**

The weakness of the article is sampling, purposive based on what inclusion criteria ? Why gender is significant different, is there any explanation or biasness. Authors can provide the tools as a supplement materials. Covid lockdown result in bad impact on dengue control, is it evidence enough by your study findings.

**Response:** Thank you so much for this observation. We have added details on inclusion criteria for purposive sampling **(Please see lines 167-174 on Page 7)**. We have provided study tools as supplementary material to this manuscript. We also very much agree with your feedback on COVID-19 impact on dengue control and our findings may help in dengue solutions, particularly among children in COVID-19 like conditions.

**Response to Reviewer 2 comments**

The research paper titled "A Primary School-Based Dengue Solution Model for Post-COVID-19 in Southern Thailand: Students' Understanding of the Dengue Solution and Larval Indices Surveillance System," by Jiraporn Jaroenpoola and colleagues, presents an intriguing and valuable model for preparedness and response in unprecedented situations. This model could be useful if it could be effectively replicated in other regions.
I have the following suggestions for enhancing the manuscript:

**Response:** Thank you so much for your constructive feedback. We have incorporated all your feedback in the revised version of our manuscript.

**Abstract**

1. The abstract should concisely summarize the manuscript’s key findings, presenting them clearly and comprehensively. Revise the abstract by highlighting the most significant outcomes and selecting the most relevant keywords. Consider removing "Aedes aegypti" and instead focus on keywords that directly reflect the main findings and themes of the study.

**Response:** Thank you. We have revised our abstract and we have also removed "Aedes aegypti" from key terms. **Please see lines 49-57 on Page 2.**

**Background**

1. Introduction to Dengue Virus Infection: A brief introduction to dengue virus infection outlines its significance as a public health concern, particularly in tropical and subtropical regions like Southern Thailand.

**Response:** Thank you so much. We have added suggested details. **Please see lines 72-80 on Page 3.**

2. Current Situation of Dengue Virus Infection: Discuss dengue infections in Thailand overall and Southern Thailand, including any recent trends or data post-COVID-19. Highlight the challenges that have emerged due to the pandemic's impact on public health systems.

**Response:** Thank you. We have added suggested details. **Please see lines 97-105 on Page 4.**

3. Need for a Dengue Solution Model: Explain why there is an urgent need for an innovative dengue solution model over another existing model, particularly in the context of primary schools. I suggest emphasising the importance of larval indices surveillance as a key component of this model and its potential to mitigate dengue transmission in the community.

**Response:** Thank you. We have added suggested details. **Please see lines 104-125 on Page 5.**

4. Justification and Knowledge Gaps: I suggest providing a rationale for the current study by identifying gaps in contrast to existing research and knowledge. Justify the need for this research by explaining how it addresses these gaps and contributes to the understanding and control of dengue in post-COVID-19 settings. Is there a decreased number of dengue cases in ASEAN countries directly linked with the movement of people or the reporting system since Singapore did not experience as with other ASEAN countries?

**Response:** Thank you. We have given details regarding. **Please see lines 138-148 on Page 6.**

**Methods**

1. Selection of Primary Schools: Justify the selection of the four primary schools included in the study. Provide criteria or reasoning that guided this selection, such as the geographical location, dengue risk levels, or previous history of dengue outbreaks.

**Response:** Thank you. Keawsan subdistrict in Nabon district has four primary schools, and all the four schools were included in the study. It was also mentioned in the introduction part that this area has been designated as a dengue risk area in southern Thailand. **Please see lines 160-162 on Page 7.**

2. How do the investigators select leader and non-leader groups?

**Response:** Thank you. The student participants were permitted to willingly choose which of the two groups they want to join and it is mentioned in the revised manuscript. **Please see lines 170-172 on Page 7.**

3. Pre-testing of the Developed Questionnaire: Elaborate on the pre-testing process of the developed questionnaire. Explain how this process ensured the reliability and validity of the data collection tool, and how it was tailored to the context of the study.

**Response:** Thank you for this comment. The suggested details have been added. **Please see lines 231-244 on Pages 10-11.**

4. Integration of the Dengue Model: Describe how the dengue solution model was integrated into the school-based setting. Discuss the steps taken to ensure that the model was both practical and adaptable to the needs of the students and the school environment.

**Response:** Thank you. We have further elaborated on the integration of dengue model. **Please see Pages 11-14.**

5. Assurance of Model Effectiveness: Outline the methods used to evaluate and validate the effectiveness of the developed and adapted dengue control model. Provide details on how the model’s success was measured and any criteria used to assess its impact.

**Response:** Thank you for this comment. We provided further details for the model effectiveness. **Please see lines 249-261 on Pages 11-12.**

**Results**

1. Presentation of Findings: Construct a 2x2 table to present the major findings of the study in a clear and concise format and tables Summarize key outcomes, focusing on the most significant results rather than getting bogged down in excessive detail.

**Response:** Thank you so much. We have designed our tables according to the journal guidelines and all tables provide important details. **Please see Tables in the revised manuscript.**

2. What was the impact of the study on the parents and the family members?

**Response:** The parents or the family members were not involved in this study, so data were not collected from them. Although the students were told to repeat the mosquito larvae survey in their respective homes, maybe when the students are carrying out the activities, they may have learnt some information about dengue control from their children, and apart from that, the training handbooks were given to the students to share with their family members and train them what they have learnt.

**Discussion**

1. Is there any previous study Comparison with Previous Studies: Compare the findings of this study with previous research conducted on similar topics. Highlight any similarities or differences and provide logical explanations for any discrepancies observed between this study and existing literature.

**Response:** Thank you so much. We have already discussed our findings with earlier studies and new studies for comparison have been added. **Please see lines 390-406 on page 20.**

**Limitations**

1. Study Limitations: Discuss the limitations of the study, including any constraints or potential biases that could affect the findings. Be transparent about these limitations to provide a balanced view of the study's strengths and weaknesses.

**Response:** Thank you for this. A section on limitation has been added to the revised manuscript. **Please see page 24.**

**Suggestions for future research, implementation challenges, and recommendations**

**Response:** Thank you for this. We have thorough revised our conclusion and these points have been highlighted. **Please see conclusion on page 24.**

**Response to Reviewer 3 comments**

The study "A primary school-based dengue solution model for post-COVID-19 in southern Thailand: Students understanding of the dengue solution and larval indices surveillance systemis" is the result of rigorous research that provides useful information for those working in the education and prevention of dengue and other communicable diseases. The researchers applied appropriate methodologies to achieve the objectives, as it is a mixed-methods study with both qualitative and quantitative methodologies. The analyses are detailed and apply relevant statistics to each case, and the results are correctly expressed. The figures and tables provide sufficient and easy-to-understand information. The sample size is adequate, and although some participants dropped out of the study, the analysis was still completed. The results adequately characterize the study population and provide evidence for the conclusions.
The statistical analysis is the best developed part of the manuscript.
The researchers propose an interesting model that is valid for dengue prevention in primary school children, however it would be useful if they described the limitations they found in developing the training of children and the recommendations to increase the impact of the model or the adjustments they would suggest to achieve percentages higher than 80% in all aspects of dengue prevention.

**Response:** Thank you so much for the positive evaluation of our manuscript.

1. Most of the references are appropriate, but I have pointed out three changes in the citation in the text (see annex). Apply reference to this statement from the introduction section: The movement of individuals over national borders is the main factor contributing to the spread of both COVID-19 and dengue infection, which increases the likelihood of a continuous outbreak.
   In the first paragraph of the discussion: This can be explained by the Aedes mosquito's diurnal adaptation to stagnant or stored water, which occasionally attracts children.
   Find the reference to the vector biology study, which determines the diurnal biting behavior for Aedes, and therefore change citation number 26.

**Response:** Thank you for bringing these important references to our attention. We have added the updated references. We have also corrected sentences highlighted by the esteemed reviewer.

We would like to thank once again the respected editor and esteemed reviewers for their constructive feedback and help, and we hope that the revised version of our manuscript will be deemed suitable for publication in this prestigious journal.

Sincerely,

Charuai Suwanbamrung ([yincharuai@gmail.com](mailto:yincharuai@gmail.com))

School of Public Health, Walailak University, Thailand
